# Ultrasmall gold nanorod-polydopamine hybrids for enhanced photoacoustic imaging and photothermal therapy in second near-infrared window

**DOI:** 10.7150/ntno.63634

**Published:** 2022-01-01

**Authors:** Wonjun Yim, Raina M. Borum, Jiajing Zhou, Yash Mantri, Zhuohong Wu, Jingcheng Zhou, Zhicheng Jin, Matthew Creyer, Jesse V. Jokerst

**Affiliations:** 1 Materials Science and Engineering Program, University of California San Diego, La Jolla, California, 92093, United States.; 2 Department of Nanoengineering, University of California San Diego, La Jolla, California, 92093, United States.; 3 Department of Bioengineering, University of California San Diego, La Jolla, California, 92093, United States.; 4 Department of Radiology, University of California San Diego, La Jolla, California, 92093, United States.

**Keywords:** miniature gold nanorod, core-shell structure, synthetic melanin, second near-infrared

## Abstract

Gold nanorods (GNRs) have attracted great interest for photo-mediated biomedicines due to their tunable and high optical absorption, high photothermal conversion efficiency and facile surface modifiability. GNRs that have efficient absorption in second near-infrared (NIR-II) window hold further promise in bio-applications due to low background signal from tissue and deep tissue penetration. However, bare GNRs readily undergo shape deformation (termed as 'melting effect') during the laser illumination losing their unique localized surface plasmon resonance (LSPR) properties, which subsequently leads to PA signal attenuation and decreased photothermal efficiency. Polydopamine (PDA) is a robust synthetic melanin that has broad absorption and high photothermal conversion. Herein, we coated GNRs with PDA to prepare photothermally robust GNR@PDA hybrids for enhanced photo-mediated theranostic agents. Ultrasmall GNRs (SGNRs) and conventional large GNRs (LGNRs) that possess similar LSPR characteristics as well as GNR@PDA hybrids were compared side-by-side in terms of the size-dependent photoacoustic (PA) imaging, photothermal therapy (PTT), and structural stability. *In vitro* experiments further demonstrated that SGNR@PDA showed 95% ablation of SKOV3 ovarian cancer cells, which is significantly higher than that of LGNRs (66%) and SGNRs (74%). Collectively, our PDA coating strategy represents a rational design for enhanced PA imaging and efficient PTT via a nanoparticle, i.e., nanotheranostics.

## Introduction

Nanotechnology has significantly advanced the development of bioimaging and light-activated phototherapy such as photoacoustic (PA) imaging and photothermal therapy (PTT) 1, 2. In PA imaging, a pulsed laser illuminates biological tissues or a contrast agent, and the absorbed light energy is converted into heat energy thereby leading to transient expansion of the absorber and generating acoustic waves detected via ultrasonic transducers 3. PA imaging is noninvasive and offers high spatial resolution, deep penetration, and fast imaging speed. It has attracted immense attention of researchers for biomedical imaging 4-6. Both endogenous (e.g., hemoglobin or melanin) and exogenous (e.g., plasmonic nanoparticles) contrast agents can be used for PA imaging to monitor specific biological processes or to improve imaging depth and quality in tissue 7.

PTT is a spatiotemporally controllable cancer treatment that has shown great promise in the cancer therapy 8. Like PA imaging, PTT is based on the conversion of light into heat, but it uses heat for thermal ablation against cancer cells. Compared to other conventional methods including radio- or chemo-therapy, PTT is attractive because of high inherent specificity, low cost, and limited invasiveness 9. For example, PTT causes limited damage to the surrounding healthy tissues because thermal effect occurs only in the presence of PTT agents 10. Currently, the research topics on photothermal theranostics has expanded rapidly, which greatly benefits from the advances made in novel nanomaterials design, including inorganic, organic, and hybrid nanoparticles 11-14.

Particularly, gold nanorods (GNRs) are widely investigated for both PA imaging and PTT due to their high absorption efficiency, photothermal conversion efficiency, and low toxicity. Moreover, the localized surface plasmonic resonance (LSPR) of GNRs is easily tunable by controlling their aspect ratio. GNRs with high aspect ratio show optical absorption in second near-infrared (NIR-II) window (1000 ‒ 1350 nm) which offers deep penetration of light, low background noises from the tissue, and maximum permission exposure (MPE) compared to NIR-I window (650 ‒ 950 nm) 15-17. However, pristine GNRs have a significant limitation: laser-induced shape deformation from thermodynamic instability. Under laser irradiation, the thermodynamic tendency to lower the surface energy makes the rod-like GNRs form nanospheres with greater stability (reduced surface area to volume ratio) 18. Specifically, it is found that a high-pulsed laser induces both point and line defects that grow into planar defects in the interior of GNRs 19. These defects consequentially convert GNR {110} facets into the energetically stable {100} and {111} facets to minimize surface energy 18. As a result, GNRs lose their NIR absorption peak due to the structural deformation thus attenuating PA signal and photothermal efficiency. Therefore, improving thermal stability of GNR could improve the potential use of GNR in photo-mediated applications.

Size reduction is one strategy that can improve photothermal stability. Recently, Emelianov and Gambhir *et al.* reported a seedless method to synthesize ultrasmall GNRs (SGNRs) 20. These SGNRs are 50% smaller than conventional large GNRs (LGNRs), but they maintained a comparable aspect ratio and demonstrated 3-fold PA enhancement due to their higher surface-to-volume ratio. More interestingly, these SGNRs were more photothermally stable than LGNRs after laser irradiation 20. Nevertheless, further GNR miniaturization for thermal stability optimization is difficult; proper synthesis relies on a wide set of factors that can influence particle growth like choice in concentration, surfactant, reducing agent, and pH. Alternatively, core-shell nanostructures can provide further improvement in photothermal stability and performance of SGNRs. Many groups have investigated a variety of materials such silica, metal oxide, and polymers as coatings for GNRs 21-24. However, the inherent properties of many of these coatings limit their use in biomedical applications. For example, polysarconsine-coated GNRs show high colloidal stability, but they have poor surface modification 25. CuO or MnO_2_-coated GNRs have facile surface tunability but suffer from cytotoxicity 26, 27. In contrast to these methods, we used polydopamine (PDA) to protect pristine GNR probe from the shape deformation. PDA coatings are robust, biocompatible, and surface functionalization 28, 29. We thus hypothesized that PDA is a pragmatic choice as a GNR shape-preserving coating for theranostic applications.

**Figure 1:**
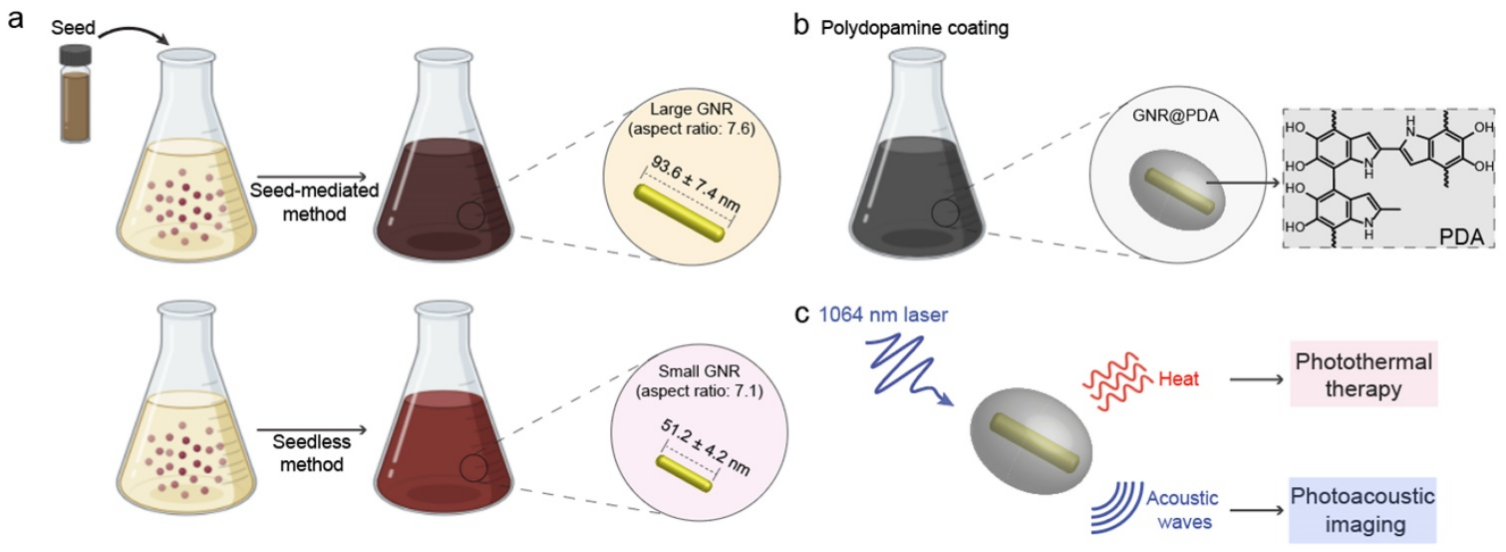
(a) Schematic of the synthesis of LGNRs (seed-mediated method, 93.6 ± 7.4 nm in length) and SGNRs (seedless method, 51.2 ± 4.2 nm in length) which have similar aspect ratio of ~7. (b) The CTAB-stabilized GNRs were replaced with HS-mPEG_2k_ to make GNR@PDA hybrids with the PDA thickness of 25 nm. (c) PA imaging and PTT of GNR@PDAs were studied using a laser irradiation with the wavelength of 1064 nm.

PDA is a synthetic melanin that is a highly crosslinked structure with broadband absorption. Its abundant catechol moieties accommodate a wide class of noncovalent interactions (e.g., hydrophobic, electrostatic, hydrogen bonding, and π‒π stackings) with diverse materials (e.g., inorganic, organic, and protein) 30, 31. We previously validated PDA coatings improve PA performance and thermal stability of pristine LGNR 32. However, a critical size limit of nanoparticles is ~ 100 nm for in vivo applications 33. For example, FDA-approved anticancer nanomedicines (e.g., doxorubicin and paclitaxel) which are in size range of 100 ~ 200 nm showed limited accumulation and penetration in tumors 34, 35, while nanomedicines with the size below 100 nm demonstrated superior penetration and retention in tumor tissues 36-38. Furthermore, photothermal performance of GNR@PDA still remains unclear. Herein, we investigated PDA-coated SGNR to improve photoacoustic and photothermal performance of the core SGNR nanoparticles. First, we synthesized LGNR and SGNR that have comparable longitudinal absorption peak in NIR-II window **(Figure 1a)**. We then coated PDA on both LGNR and SGNR to investigate both PA efficiency and photothermal conversion efficiency **(Figure 1b-c)**. Finally, human ovarian adenocarcinoma (SKOV3) cancer cells were used to evaluate photothermal therapeutic efficiencies between LGNR (66%), SGNR (74%), and SGNR@PDA (95%). Together, these size-dependent and PDA coating effects provide comprehensive insight in designing stable GNR nanoprobes for photo-mediated nanomedicine applications.

## Materials and Methods

Gold(III) chloride hydrate (HAuCl_4_), cetyltrimethylammonium bromide (CTAB), sodium borohydride (NaBH_4_), hydroquinone, silver nitrate (AgNO_3_), dopamine hydrochloride, bicine, resazurin, and McCoy's 5A medium were purchased from Sigma-Aldrich (Atlanta, GA, USA). Methoxy poly(ethylene glycol) thiol (HS-mPEG) with molecular weights (M_w_) of 2k, 5k, 10k, and 20k were purchased from Nanocs. Dulbecco's modified Eagle's medium (DMEM) was purchased from Thermo Fisher. Calcein AM (4 mM) dissolved in DMSO was purchased from Biotium. Propidium iodide (≥ 95%, PI) was purchased from Combi-Blocks (San Diego, CA, USA). All reagents were used without further purification. Deionized water (18.2 MΩ·cm) purified with a Milli-Q Academic water purification system was used to make aqueous solutions.

### Synthesis of Large GNR

We used a seed-mediated growth to synthesize regular size of GNR nanoparticles with a high aspect ratio of 7.1 39. A seed solution was prepared by mixing an aqueous solution of 5 mL of CTAB (0.2 M) and 5 mL of HAuCl_4_ (0.5 mM). Then, 600 µL of NaBH_4_ (10 mM) was quickly injected to the aqueous solution under vigorous stirring at 1200 rpm. After 15 s of vigorous reaction of NaBH_4_, the stir bar was removed, and the seed solution was incubated in a water bath at 30 °C for 1 h.

A growth solution was made by adding sequentially aqueous solution of 5 mL of CTAB (0.2 M), 5 mL of HAuCl_4_, and 60 µL of AgNO_3_ (0.1 M). Then, 325 µL of hydroquinone (0.1 M) was injected under vigorous stirring at 1200 rpm. The color of the growth solution changed from yellow to transparent; 165 µL of the seed solution was then injected to the growth solution under vigorous stirring at 1200 rpm for 15 s. The solution was incubated in water bath at 30 °C for 12 h before being purified with centrifugation at 7,500 *g* for 10 min. After centrifugation, CTAB-stabilized LGNRs were redispersed in 2 mL of deionized water.

### Synthesis of Ultrasmall GNRs

We used a seedless method to synthesize ultrasmall of GNR nanoparticles with a high aspect ratio of 7.7 20. Briefly, 5 mL of CTAB (0.2 M), 4 mL of HAuCl_4_ (0.5 mM), 30 µL of AgNO_3_ (0.1 M), 20 µL of HCl (1.0 M), and 530 µL of hydroquinone (0.1 M) were sequentially added. The color of solution was bright yellow and stirred at 1000 rpm for 15 min. Finally, 20 µL of fresh NaBH_4_ (10 mM) solution (prepared within 10 min of use and stored on ice) was quickly injected under vigorous stirring at 1100 rpm. After 15 s of NaBH_4_ reaction, the solution was incubated in water bath at 30 °C for 12 h before being purified with centrifugation at 20,000 *g* for 15 min. After centrifugation, CTAB-stabilized SGNR was redispersed in 2 mL of deionized water.

### Surface Modification of Large and Ultrasmall GNRs

CTAB on the surface of LGNR and SGNR was replaced with HS-mPEG (M_w_, 2k Da) via ligand exchange. Both CTAB-stabilized LGNR and SGNR were dispersed in 2 mL of deionized water and added to 4 mL of HS-mPEG (5 mg/mL, 1mM) under generous stirring at 900 rpm for 12 h. The PEGylated LGNR was purified by centrifugation at 7,500 *g* for 10 min while the PEGylated SGNR was purified by centrifugation at 20,000 *g* for 15 min to remove excess of HS-mPEG in supernatant. The PEGylated LGNR and SGNR were redispersed in 2 mL of water for future use.

### Synthesis of PDA Coated GNRs

The 200 µL of LGNR solution was dispersed in 2 mL of bicine buffer (10 mM, pH 8.5). Then, 200 µL of dopamine solution (4 mg/mL) was added under vigorous stirring at 1300 rpm for 12 h. Similarly, 200 µL of SGNR solution was dispersed in 2 mL of bicine buffer (10 mM, pH 8.5) followed by 200 uL of dopamine solution (4 mg/mL) under vigorous stirring at 1300 rpm for 12 h. The color of solution was dark-brown, and the resulting product was purified by centrifugation at 7,500 *g* for 10 min. Both LGNR@PDAs and SGNR@PDAs were redispersed in water for future use.

### Cell Culture and Preparation

Human ovarian adenocarcinoma (SKOV3) cells were cultured in complete McCoy's 5A medium (1.5 mM L-glutamine, and 2.2 g/L sodium bicarbonate and supplemented with 10% fetal bovine serum and 1% penicillin/streptomycin). Cell cultures were incubated under 5% CO_2_ at 37 °C. Cultures were given at least three passages before they were used for experiments. Cells were passaged from 75 to 80% confluency using 0.25 % Trypsin-EDTA. Photobleaching was used to confirm healthy and dead cells.

### Cytotoxicity Assay

SKOV3 cells were seeded overnight in a 96-well plate at a concentration of 20,000 cells/well. After seeding, LGNR, SGNR, and SGNR@PDA were incubated with the SKOV3 cells at equal concentration (10 μM) for 24 h. Each well was then washed three times with cold phosphate-buffer saline (PBS) to remove the free nanoparticles. A resazurin assay was used to analyze cytotoxicity of LGNR, SGNR, and SGNR@PDA following a general protocol: After cells were incubated with resazurin for 4 hours, cell viability was calculated by measuring the subtracted background absorbance of each cell at 600 nm from resazurin absorbance at 570 nm. The absorbance of experimental wells was compared to that of the controlled well including healthy and dead cells. All experiments were performed in triplicate, and the results were averaged.

### In Vitro PTT in NIR-II Window

SKOV3 cells were seeded in a 96-well plate at 20,000 cells/well overnight. Each sample well was spaced out by an empty well to rule out the interference of heat transfer from the other wells during the laser irradiation. 100 µL of LGNR, SGNR, and SGNR@PDA at the same concentration in 1.0 µL in McCoy's 5A medium was incubated with SKOV3 cells for 24 h. Each sample well was then exposed to a 1064 nm laser for 10 min. After NIR-II laser irradiation, samples were incubated for another 12 h, and the photothermal cytotoxicity was calculated by using a resazurin assay as described above. All experiments were done in triplicate, and the results were averaged.

### Cell staining with Calcein AM and Propidium Iodide Dyes

Calcein AM and Propidium Iodide (PI) were used to stain the living and dead cells before and after NIR-II laser irradiation following a general protocol. SKOV3 cells were seeded in a 24-well plate (50,000 cells/well) overnight and then incubated with LGNR, SGNR, and SGNR@PDA with the same concentration of 1.0 μM for 24 h. After NIR-II laser irradiation, each sample well was incubated for 12 h, and cells without laser irradiation was used as a control. 900 µL of mixture solution of calcein AM (2 µM) and PI (6 µM) was added to stain the SKOV3 cells. The fluorescence images of each sample well were taken by using an EVOS FL fluorescence microscope after washing with PBS.

### Instrumentations

The diluted GNR and GNR@PDA samples were dropped onto copper grids for transmission electron microscopy (TEM) measurements. TEM images were examined with a JEOL JEM 1400 Plus operating at 80 kV. The TEM images were taken via a Gatan 4k digital camera. Electron-dispersive X-ray spectroscopy (EDX) samples were examined using a Thermo Fisher Talos 200X operating at 200 kV. Scanning TEM (STEM) images were examined, and EDX maps were acquired by using a Thermo Scientific software. The hydrodynamic diameter and the zeta potential of each sample were measured by using a Malvern Instrument Zetasizer ZS 90; 100 µL of each sample was diluted in 900 µL of distilled water. Ultraviolet‒visible‒NIR (UV‒vis‒NIR) absorption spectra of each sample were measured using a PerkinElmer UV‒vis‒NIR spectrophotometer. Absorbance was read from 400 nm to 1350 nm with a step size of 3 nm. The inductively coupled plasma mass spectrometry (ICP-MS) analysis was performed using a Thermo Scientific iCAP RQ ICP‒MS in the Environmental and Complex Analysis Laboratory at UC San Diego. Samples were digested using *aqua regia* and prepared in 10 mL of 4% HNO_3_. Regression analysis (R-squared) was calculated by using Microsoft Excel.

### Photoacoustic Imaging of LGNR, SGNR and SGNR@PDA

A VisualSonics Vevo 2100 LAZR imaging system was used for PA imaging at 1064 nm. Samples were imaged using a 21 MHz-centered LZ 250 transducer. The NIR-II laser was calibrated and optimized before the sample measurement. The specimens were positioned at a depth of 1 cm from the transducer. Temperature-dependent PA imaging was performed in the water bath at different temperatures (e.g., 4, 10, 20, 37, and 45 °C). Real-time temperature of water bath was measured using a Hg thermometer. Ice and hot plate were used to cool or heat the water bath. All PA data were processed using Image J software 40. The average value and standard deviation of the PA intensity were calibrated based on the five regions of interest per tube. The R-squared and slope of PA intensity was calculated using GraphPad Prism software.

### Photothermal Conversion Efficiency of LGNR, SGNR, and SGNR@PDA

Typically, 1 mL of LGNR, SGNR, and SGNR@PDA samples at the same concentration was placed in a quartz cuvette to measure photothermal conversion efficiency. We used a 1064 nm laser with the power density of 1.0 W/cm^2^. Real-time temperature was monitored every 30 s by using a FLIR C5 camera. Each sample was irradiated with 1064 nm for 30 min. The laser turned off after 30 min of irradiation, and the cooling rate was carefully recorded to calculate photothermal conversion efficiency following equation **(see Supporting Information)**.

## Results and Discussion

### Synthesis and characterization of SGNR@PDA

**Figure 2:**
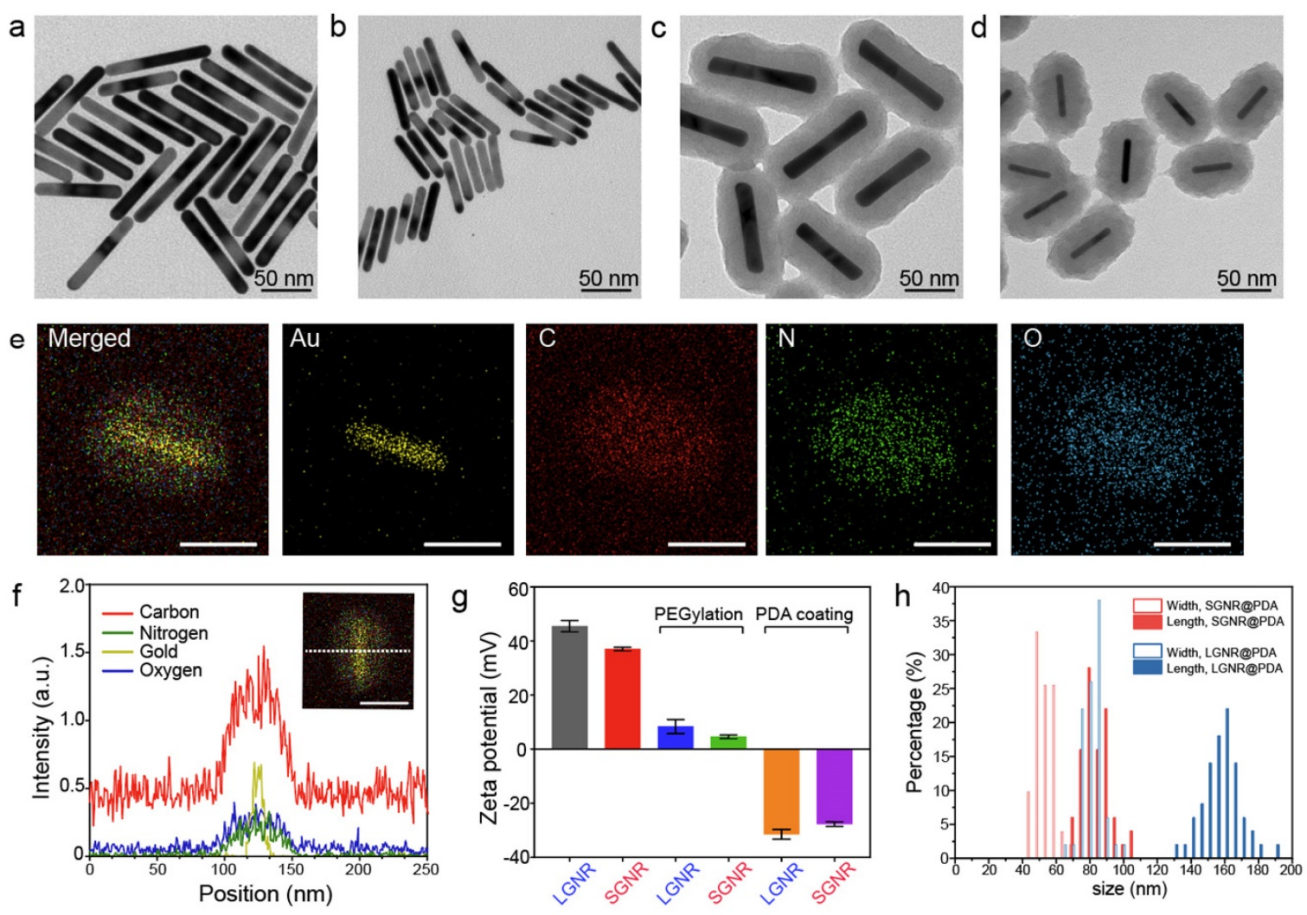
PDA-coated large and ultrasmall GNRs. TEM images of (a) LGNRs, (b) SGNRs, (c) LGNR@PDA, and (d) SGNR@PDA. Same PDA thickness about 25 nm was coated on both LGNRs and SGNRs. (e) EDX mapping of SGNR@PDAs shows the core-shell nanostructure of SGNR-polydopamine nanohybrids. The scale bar represents 50 nm. (f) EDX line scanning of SGNR@PDAs. The inset image indicates the location of SGNR@PDAs that were used to analyze the signal intensity of Au, C, N, and O. (g) Zeta potential of LGNR and SGNR before and after PEGylation and PDA coating. Zeta potential of CTAB-stabilized GNRs decreased after ligand exchange, and the surface charge of PEGylated GNRs further decreased when coated with negatively charged PDA. The error bar represents standard deviation of five measurements. (h) Histogram of SGNR@PDA and LGNR@PDA indicates that the length of LGNR@PDA is 2-fold larger than that of SGNR@PDA. Fifty nanoparticles were used to calibrate the width and length of the GNR@PDA nanohybrids.

GNRs have long been synthesized via seed-mediated growth methods 41, 42. We used a conventional seed-mediated method to make LGNRs with 93.6 ± 7.4 nm (length) ×12.4 ± 1.4 nm (width) dimensions and an aspect ratio of 7.6 **(Figure 2a)** 41. To miniaturize GNRs while maintaining high aspect ratio, the size and number of gold seeds became a limiting factor 20. We therefore used a seedless method to make SGNRs with 51.1 ± 4.2 nm (length) × 7.2 ± 1.2 nm (width) dimensions and an aspect ratio of 7.1 **(Figure 2b)**. Both LGNRs and SGNRs were stabilized with CTAB and had a positive surface charge (45.5 ± 2.1 mV for LGNRs, and 37.0 ± 0.6 mV for SGNRs). The formation of PDA coating is easily induced by auto-oxidation of dopamine under basic conditions (pH > 7.5). Under weak alkaline conditions, dopamine undergoes successive oxidation, intramolecular cyclization, oligomerization, and then self-assembly into polymerized PDA 43. Taking advantages of strong adhesive properties of PDA, we uniformly coated PDA on the surface of both LGNRs and SGNRs in bicine buffer (pH 8.5). TEM images show monodispersed LGNR@PDA and SGNR@PDA nanohybrids with same 25 nm PDA coating thickness **(Figure 2c-d).** The coating thickness was readily tunable from 5 nm to 50 nm by adjusting the amount of dopamine feed (4 mg/mL) in the reactions **(Figure S1)**. EDX mapping and high-angle annular dark-field imaging (HAADF) confirmed the core-shell nanostructures of SGNR-polydopamine hybrids composed of Au, C, N, and O **(Figure 2e** and **Figure S2)**. Furthermore, EDX line scanning indicated that SGNR nanoparticles were covered by carbon—the main component of PDA (**Figure 2f**). Adding dopamine directly to CTAB-stabilized LGNRs and SGNRs led to particle aggregation due to strong electrostatic interactions between positively charged GNRs and negatively charged dopamine. Therefore, we replaced CTAB with a methoxy PEG thiol of M_w_ 2k Da (HS-mPEG) to accommodate stable PDA coating **(Figure S3)**. Using strong Au-thiol bonding formation 44, we successfully exchanged the surfaces of both LGNRs and SGNRs with HS-mPEG, where the zeta potential of LGNRs and SGNRs decreased to 8.3 ± 2.6 mV and 4.6 ± 0.6 mV, respectively **(Figure 2g)**. PEGylated LGNRs and SGNRs carry more negative charges after PDA coating due to the phenolic hydroxyl groups on PDA. Histogram of SGNR@PDA and LGNR@PDA sizes as measured by TEM shows that the size of LGNR@PDA nanohybrids is two-fold larger than that of SGNR@PDA nanohybrids **(Figure 2h)**.

### Photoacoustic performance of SGNR@PDAs

**Figure 3:**
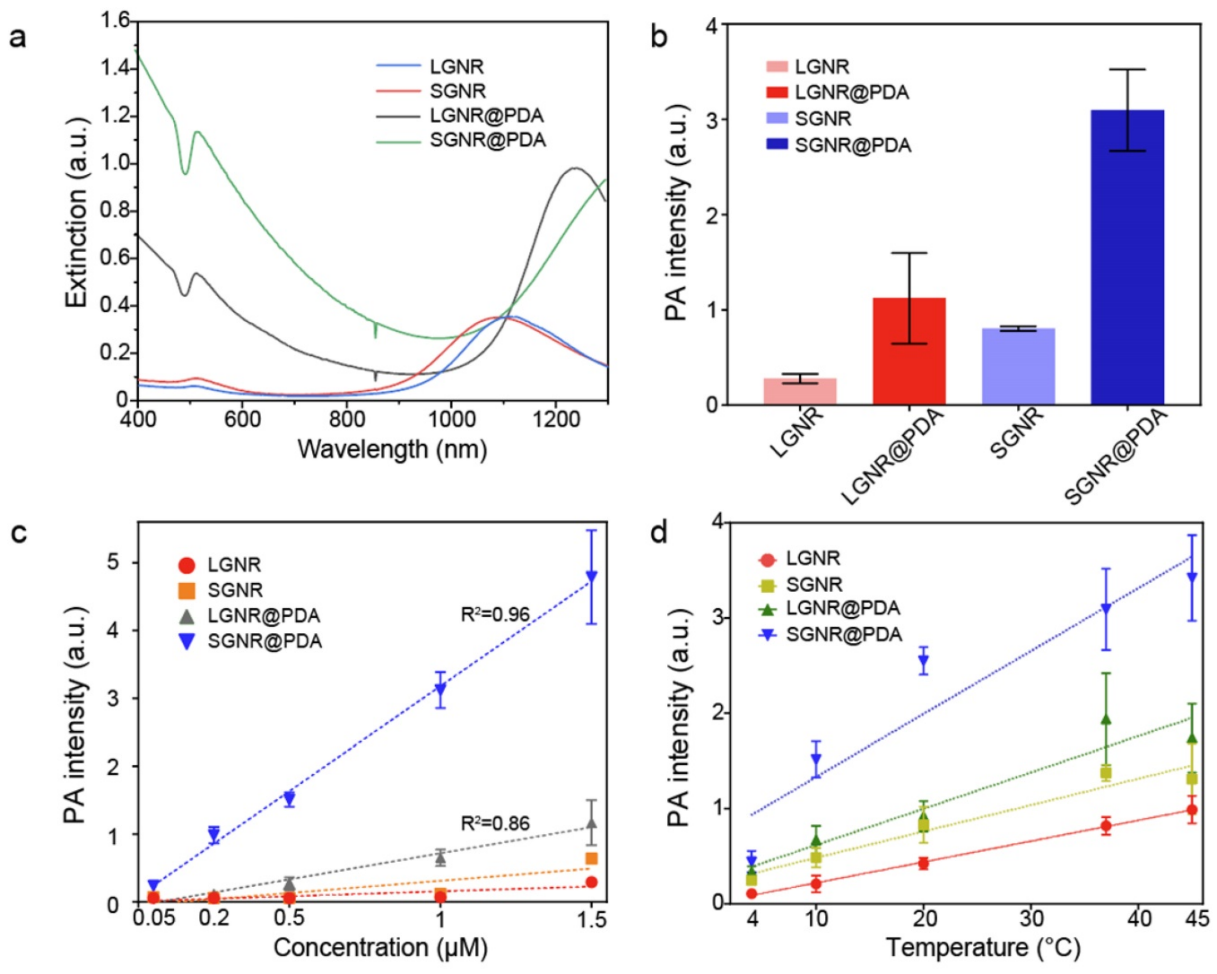
PA imaging of GNR@PDAs (a) UV‒vis‒NIR absorption spectra of LGNR, SGNR, LGNR@PDA, and SGNR@PDA. Longitudinal absorption peaks of LGNR and SGNR redshifted after PDA coating due to the increased refractive index. Molar concentrations of pristine GNR and GNR@PDA are the same. (b) PA intensity of LGNR, LGNR@PDA, SGNR, and SGNR@PDA under NIR-II (1064 nm) laser irradiation. PDA coating improved PA performance of core GNR particles. (c) PA intensity of LGNR, SGNR, LGNR@PDA, and SGNR@PDA with the elevated sample concentration under NIR-II (1064 nm) laser irradiation. SGNR@PDA showed 4-fold higher PA signal generation than pristine SGNR. (d) PA intensity of LGNR, SGNR, LGNR@PDA, and SGNR@PDA measured in different temperature (e.g., 4, 10, 20, 37, and 45 °C). Slopes of temperature versus PA intensity of LGNR, SGNR, LGNR@PDA, and SGNR@PDA are 0.022, 0.027, 0.038, and 0.066, respectively. The error bars represent the standard deviation of five regions of interest.

When LSPR nanoparticles are in solution, light is mainly absorbed by the nanoparticles rather than the solvent. The plasmonic SGNR nanoparticles convert absorbed light energy into heat generating acoustic energy into the surrounding medium. Both heat and stress leak out from the nanoparticles during nanosecond-pulsed laser irradiation due to high thermal conductivity of gold and nanometric volume of the particles 45. Therefore, PA signal is determined by optical absorption of nanoparticles as well as heat transfer rate from gold to water 46. UV‒vis‒NIR absorption spectra showed that LGNR had two absorption peaks at 504 nm and 1076 nm that correspond to the transverse and longitudinal LSPR, respectively. Likewise, SGNR exhibited two absorption peaks at 512 nm and 1064 nm, validating that GNR aspect ratio determines its LSPR absorption peaks **(Figure 3a** and **Figure S5)** 47. The longitudinal peaks of both GNRs were significantly redshifted when PDA was coated due to the increased refractive index (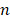) from the surrounding medium (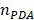 ≈ 1.7, and 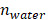
≈ 1.3) 48.

We illuminated optical density (OD)-matched GNR solutions at 1064 nm to measure PA performance of LGNR and SGNR. Consistent with a previous study 20, SGNR (OD ≈ 1 at 1064 nm) showed 3-fold higher PA intensity than LGNR (OD ≈ 1 at 1064 nm) because of its higher surface-to-volume ratio that can improve heat transfer **(Figure 3b)** 47. We then used inductively coupled plasma mass spectrometry (ICP-MS) to match each GNR and GNR@PDA concentrations based on the number of Au ions in each sample **(Figure S4)**. As a result, optical extinctions of both LGNR@PDA and SGNR@PDA were higher than pristine LGNR and SGNR due to the absorbance of PDA coating and the increased size of GNR-melanin hybrid nanoparticles. The increase in absorption intensity is important because PA signal is directly a function of the optical and thermoelastic properties of nanoparticles according to the thermoelastic expansion model (**equation 1**) 3.


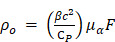

(1)

where, 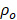, 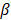, 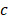, 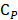, 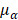, and 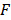 are pressure gradient, thermoelastic expansion coefficient, speed of sound in the medium, specific heat capacity, absorption coefficient, and laser fluence, respectively.

PDA coating further improved PA performance of both LGNR and SGNR **(Figure 3b)**. LGNR@PDA and SGNR@PDA showed three-fold higher PA signals than their corresponding pristine LGNR and SGNR. Moreover, SGNR@PDA showed 3-fold higher PA intensity than LGNR@PDA due to the inherent PA enhancement from the core SGNRs. This significant improvement in PA performance of GNR@PDAs can be explained by three parameters: optical absorption, cross-sectional area, and thermal confinement. First, the melanin-like PDA coating improved optical absorption of GNR as observed in **Figure 3a**. Consequently, GNR@PDAs can absorb more photons than pristine GNR and therefore generate more acoustic energy. Second, the cross-sectional area of GNR@PDA, as measured by electron microscopy, became larger because the PDA coating itself can absorb light. Lastly, thermal confinement obtained from the PDA shell leads to higher PA signal. The heat capacity of water (C_water_ ≈ 4.2) is 2.5 times higher than that of PDA (C_PDA_ ≈ 1.6) which means that the GNR surrounded by water requires higher energy to heat versus GNR surrounded by a PDA shell 49. Furthermore, the thermal conductivity of water (*K*_water_ ≈ 0.59) is 4 times larger than that of PDA (*K*_PDA_ ≈ 0.13) indicating that heat can readily accumulate in GNR@PDAs due to the reduced thermal conductance 49. Collectively, the PDA coating gave both LGNR and SGNR higher thermoelastic expansions than GNR alone to significantly increase PA signals.

PA signal generation is also dependent upon other factors including particle concentration and thermal expansion of surrounding solvent. As optical extinction of nanoparticles is linearly proportional to particle concentration, a linear increase in PA signal was observed with elevated concentrations **(Figure 3c)**. Importantly, SGNR@PDA at the concentration of 0.2 µM still showed high PA signal which was 15-fold higher than that of SGNR **(Figure 3c)**. The thermal expansion coefficient is an inherent material-based property, and PA signal is generated by a sum of the thermal expansion of the nanoparticle and its surrounding solvent. During nanosecond-pulsed laser irradiation, heat is not only confined within the nanoparticles but also diffused into the solvent, leading to a shell-like layer of the solvent around the nanoparticles with the increased temperature 3. To study the thermal expansion of GNR@PDAs, we measured their PA signals in different temperatures: 4, 10, 20, 37, and 45 °C. Since water was the most dense at 3.98 °C, thermal expansion of water became very low 46. That is, PA signal at 3.98 °C is mainly due to the thermal expansion of nanoparticles. LGNR, SGNR, LGNR@PDA, and SGNR@PDA nanoparticles showed the lowest PA signals at 4 °C indicating that the thermal expansion of the solvent around the nanoparticles is involved in PA signal generation **(Figure 3d)**. Importantly, the PA signal from SGNR@PDA at 10 °C significantly increased (~3 times higher than that at 4 °C) while PA signal of SGNR alone remained low. This result indicates that thermal confinement of PDA shell contributed to thermal expansion of SGNR@PDA **(Figure 3d)**.

The PA signals of LGNR, SGNR, LGNR@PDA, and SGNR@PDA linearly increased with increased temperature of the solvent. More importantly, the Grüneisen parameter of LGNR, SGNR, LGNR@GNR, or SGNR@PDA was measured based on the slope of temperature versus PA intensity because the Grüneisen parameter is a function of the thermal expansion coefficient and specific heat capacity, which are temperature-dependent 3, 46. The Grüneisen parameter (dimensionless) of SGNR@PDA (0.066) was 2.4-fold higher than that of SGNR (0.027). Likewise, Grüneisen parameters of LGNR@PDA (0.038) was 1.7-fold higher than that of LGNR (0.022). These results confirm that the PDA coating can improve the thermal conversion efficiency of core GNR nanoparticles.

### Photothermal performance of SGNR@PDAs

**Figure 4:**
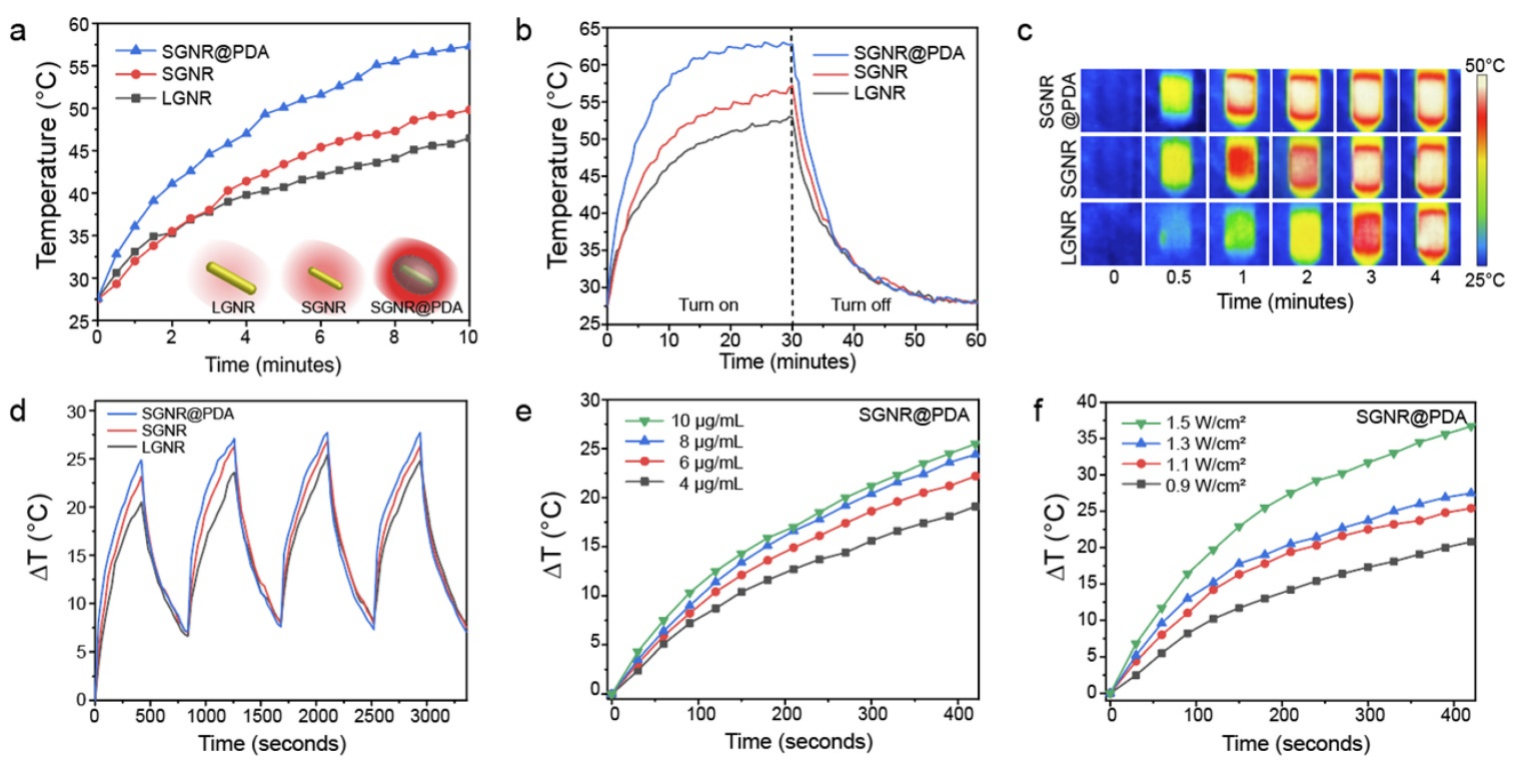
(a) Schematic illustration of the photothermal performance of LGNR, SGNR, and SGNR@PDA triggered by NIR-II (1064 nm) laser. (b) Temperature curves of LGNR, SGNR, and SGNR@PDA for 1 h under NIR-II (1064 nm) laser irradiation, showing that the temperature of SGNR@PDA largely increased compared to SGNR and LGNR. (c) Thermal images of LGNR, SGNR, and SGNR@PDA irradiated at 1064 nm. (d) Temperature changes of LGNR, SGNR, and SGNR@PDA under repeated “on and off” laser irradiation for four times cycle. (e) Temperature changes of SGNR@PDA with the elevated concentrations (e.g., 4, 6, 8, and 10 µg/mL) for 7 min irradiation. (f) Temperature changes of SGNR@PDA at different power densities (0.9, 1.1, 1.3, and 1.5 W/cm^2^) for 7 min irradiation. Temperature curves of SGNR and LGNR are shown in **Figure S8**.

Next, we examined the photothermal performance of LGNR, SGNR, and SGNR@PDA in NIR-II using a laser of 1064 nm for excitation. The skin-tolerance threshold set by the America Standards Institute is MPE of 1.0 W/cm^2^ for 1064 nm laser (ANSI Z136.1-2007); we therefore chose a 1064 nm laser power of 1.0 W/cm^2^ to evaluate the photothermal properties of GNR-melanin nanohybrids. **Figure 4a** shows that the temperature of LGNR, SGNR, SGNR@PDA at the same concentration of 10 µg/mL increased to 46.5 °C, 49.8 °C and 57.3 °C, respectively within 10 min; temperature change of water was negligible. Photothermal conversion efficiency is the capacity of nanoparticle to convert laser energy into heat. The laser turned off after 30 min of irradiation, and the cooling rate was recorded every 30 seconds to measure heat transferring from the nanoparticles to surroundings. Compared to other agents such as GNR@Ag (28.8%), Au@Metal-Organic Framework (30.2%) 50, 51, the SGNR@PDA showed high photothermal conversion efficiency (40%) at 1064 nm. This value was also higher than SGNR (30%) and LGNR (27%) **(Figure 4b)**. Furthermore, the photothermal conversion efficiency of PDA nanoparticle was 18% at 1064 nm indicating that GNR-melanin nanohybrids can offer outstanding photothermal performance (**Figure S6**). Superior photothermal efficiency of SGNR@PDA results from a higher optical absorption, large surface area of PDA, and improved thermal conversion efficiency (Grüneisen parameter) due to the thermal confinement of the PDA shell. Likewise, photothermal conversion efficiency of LGNR@PDA was 4% higher than LGNR **(Figure S7)**. The thermal images also showed SGNR@PDA rapidly increased temperature to 50 °C within one minute compared to SGNR and LGNR, which required three to four minutes to achieve a similar temperature **(Figure 4c)**.

SGNR@PDAs showed excellent conversion stability in four successive cycles confirming its good photostability in the NIR-II window **(Figure 4d)**. In addition, SGNR@PDAs did not show any disassembly of PDA coating or morphology changes after four successive cycles of laser irradiation **(Figure S9)**. The PDA coating can improve photothermal stability of GNR during laser irradiation because gold atoms on the surface of GNR@PDAs are tightly immobilized because of the highly crosslinked PDA shell and π-stacking structure 32. Therefore, the PDA shell serves as physical barrier while the atomic rearrangement in the GNR requires more energy. On the other hand, pristine GNRs alone cannot afford extensive heating 32. We further studied the photothermal property of SGNR@PDA in different particle concentrations and power densities. The temperature changes (ΔT) showed that the temperature increase was linearly proportional to the particle concentration. For example, ΔT of SGNR@PDA was 19.1 °C, 22.2 °C, 24.4 °C, and 25.5 °C for concentrations of 4, 6, 8, and 10 μg/mL after 7 min of NIR-II laser irradiation at the same power density of 1.0 W/cm^2^ **(Figure 4e)**.

The ΔT of SGNR@PDA also corresponded to the laser power density. For example, the temperature of SGNR@PDA increased to 20.8 °C, 25.4 °C, 27.5 °C, and 36.7 °C at the laser power densities of 0.9 W/cm^2^, 1.1 W/cm^2^_,_ 1.3 W/cm^2^_,_ 1.5 W/cm^2^ at the fixed concentration of 10 μg/mL **(Figure 4f)**. Collectively, these findings corroborated distinctive thermophilic characteristics and high photothermal conversion efficiency of SGNR@PDA highlighting its potential use in therapeutic agent for cancer treatment.

### Photothermal treatment of SGNR@PDA in vitro

**Figure 5:**
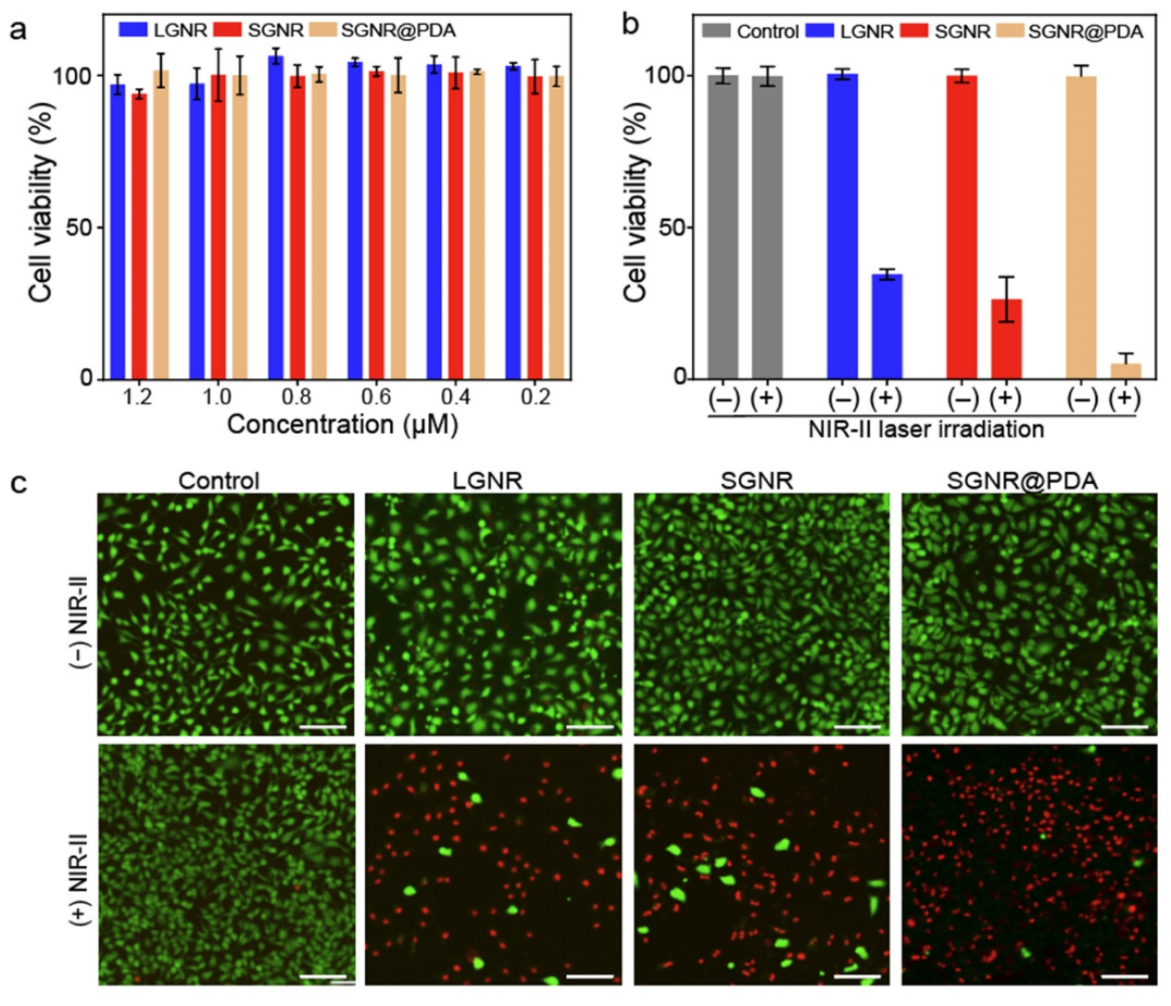
Photothermal performance of SGNR@PDA on SKOV3 cancer cell. (a) Cell viability of SKOV3 cells after co-incubated for 24 h with LGNR, SGNR, and SGNR@PDA at different concentrations. (b) Cell viability of SKOV3 cells before and after photothermal treatment (1.0 W/cm^2^ for 1064 nm, 10 min) at the same concentration of LGNR, SGNR, and SGNR@PDA (1.0 µM), indicating that SGNR@PDAs showed high photothermal-induced cell ablation compared to LGNR and SGNR. SKOV3 cells without injecting nanoparticles (referred as Control) were exposed to the laser, showing that there was no involvement of cell medium to kill the SKOV3 cells. The error bar represents the standard deviation of three measurements. (c) Fluorescence images of Control, LGNR, SGNR, and SGNR@PDA stained with Calcein AM and PI before and after laser irradiation at 1064 nm (1.0 W/cm^2^ for 1064 nm, 10 min). Photobleaching was used to confirm healthy and dead SKOV3 cells (**Figure S11**). The scale bar indicates 100 µm.

We investigated the PTT efficacy of the SGNR@PDA at the NIR-II window *in vitro*. SKOV3 cancer cells were incubated with LGNR, SGNR, or SGNR@PDA with different concentrations from 0.2 µM to 1.2 µM for 24 h. After co-incubation for 24 h, the cell viabilities of SKOV3 were greater than 90% indicating that both PEGylated GNRs and GNR@PDAs had negligible cytotoxicity **(Figure 5a)**. We then studied photothermal ablation toward SKOV3 cells when they were incubated with LGNR, SGNR, or SGNR@PDA at the same concentration (1.0 µM). Importantly, SGNR@PDA showed effective cell ablation (95%) after 10 min of laser irradiation at 1064 nm while cell ablations from LGNR and SGNR were 66% and 74%, respectively **(Figure 5b)**. SKOV3 cells without nanoparticles retained high cell viability before (100%) and after (99%) laser irradiation validating that LGNR, SGNR, and SGNR@PDA are the main factors to elicit photothermal ablation of SKOV3 cells **(Figure 5b)**.

The photothermal treatment from LGNR, SGNR, and SGNR@PDA was further studied and visualized via live-dead cell fluorescent stanning. Calcein-AM (green fluorescence for live cells) and PI (red fluorescence of dead cells) were used in these experiments. Strong green fluorescence was observed when SKOV3 cells were incubated with LGNR, SGNR, or SGNR@PDA before NIR-II laser irradiation **(Figure 5c)**. After 10 min of NIR-II laser irradiation, SKOV3 cells treated with LGNR, SGNR, or SGNR@PDA showed red fluorescence. SKOV3 cells incubated with SGNR@PDA showed strong red fluorescence, which corresponds to quantitative data: The cell ablation of SGNR@PDA was 95% **(Figure 5b-c)**. The SKOV3 cells alone were irradiated with NIR-II laser for 10 min to validate the lack of involvement of the laser toward the photothermal ablation; strong green fluorescence was observed before and after laser irradiation **(Figure 5c)**. These results indicated that the NIR-II laser alone could not cause cell death, and photothermal performance of SGNR significantly improved when PDA was coating due to higher temperature induced from the PDA shell. Finally, the colloidal stability of SGNR@PDA was investigated in different media (e.g., 10 mM, HCl, 10 mM NaOH, 10 mM NaCl, DMEM with 10% fetal bovine serum, and DMEM with the human serum of 10%). DLS data showed that SGNR@PDA maintained its GNR-melanin assemblies and showed negligible aggregation under those conditions (PDI ~ 0.1) **(Figure S10)**. These results indicate that PDA coating is highly intact and stable under biological conditions. Therefore, our PDA coating strategy can improve photothermal performance over the SGNR probe with excellent colloidal stability.

Although proof-of-concept, this work invites more studies to improve the viability of melanin-GNRs for anticancer therapy *in vivo*. One limitation of this work is lack of tumor targeting *in vivo*. In a mouse model, the photothermal performance of LGNR, SGNR, and SGNR@PDA species could be less efficient due to tissue thickness, light scattering, and absorbance from the tissue. More importantly, we do show higher photothermal performance of SGNR@PDA than SGNR due to improved optical absorption, and higher thermal conversion efficiency due to the thermal confinement of PDA shell. Future work will include photothermal treatment of SGNR@PDA in mouse model for clinical application of NIR-II PTT.

## Conclusion

Plasmonic nanomaterials, especially GNRs, hold great promise in photo-mediated applications due to their tunable and high absorption efficiency. However, anisotropic nanostructures under the laser irradiation become unstable, losing their unique optical properties. In this study, we synthesized both large GNR and ultrasmall GNR to investigate 'size-effect' and further synthesized GNR-melanin hybrids (i.e., GNR@PDA) to study 'PDA coating effect' altogether. SGNRs showed higher PA performance over large GNR due to high surface-to-volume ratio. Furthermore, SGNR@PDA showed three-fold higher PA signal than core SGNR nanoparticles and 10% higher photothermal efficiency over SGNR due to improved optical absorption and 2.4-fold higher thermal conversion efficiency. These SGNR@PDA nanohybrids also elicited effective photothermal ablation of SKOV3 cancer cells (95%) which was significantly higher than LGNR (66%) and SGNR (74%). These findings indicate high potential use of the PDA coating strategy in developing photo-mediated biomedicines in NIR-II window and expand our understanding photothermal conversion mechanism occurring in hybrid nanostructures.

## Supplementary Material

Supplementary figures.

## Abbreviations

PA: photoacoustic
PTT: photothermal therapy
NIR-II: second near-infrared
GNRs: gold nanorods
PDA: polydopamine
GNR@PDAs: polydopamine-coated gold nanorods
UV‒vis‒NIR: ultraviolet‒visible‒NIR
HAADF: High-angle annular dark-field imaging
ICP-MS: inductively coupled plasma mass spectrometry
LSPR: localized surface plasmonic resonance
SKOV3: human ovarian adenocarcinoma
DLS: dynamic light scattering
PDI: polydispersity index
EDX: Electron-dispersive X-ray spectroscopy
TEM: transmission electron microscopy
