# Supplementary material for: Ultrasmall gold nanorod-polydopamine hybrids for enhanced photoacoustic imaging and photothermal therapy in second near-infrared window
Source: Nanotheranostics. 2022 Jan 1;6(1):79–90. doi: 10.7150/ntno.63634 (PMC8671965; doi:10.7150/ntno.63634)
Supplement: Supplementary file 1 — Supplementary figures. [file ntnov06p0079s1.pdf]

Supporting information for

Ultrasmall gold nanorods-polydopamine hybrids for enhanced photoacoustic imaging and photothermal therapy in second near-infrared window

Wonjun Yim[1], Raina M. Borum[2], Jiajing Zhou[2], Yash Mantri[3], Zhuohong Wu[2], Jingcheng Zhou[2], Zhicheng Jin[2], Matthew Creyer[2], and Jesse V. Jokerst[1,2,4,*]

1. Materials Science and Engineering Program, University of California San Diego, La Jolla, California, 92093, United States
2. Department of Nanoengineering, University of California San Diego, La Jolla, California, 92093, United States
3. Department of Bioengineering, University of California San Diego, La Jolla, California, 92093, United States
4. Department of Radiology, University of California San Diego, La Jolla, California, 92093, United States

* Corresponding authors: jjokerst@ucsd.edu

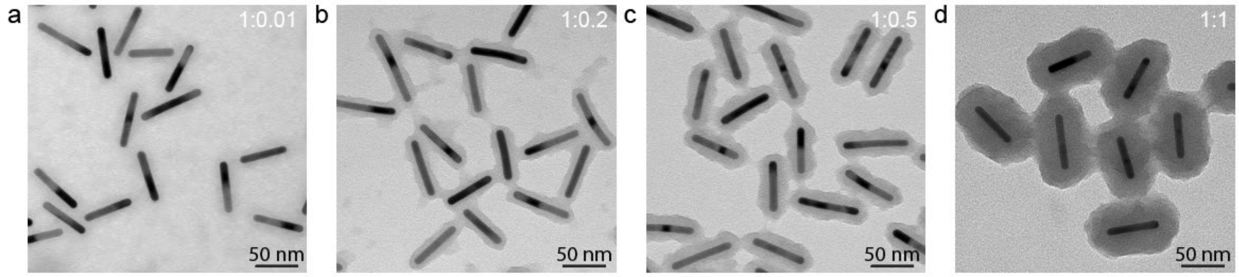

Figure S1.
TEM images with different PDA shell thicknesses: (a) 0, (b) 5, (c) 10, and (d) 25 nm. PDA thickness is controllable by adding different amount of dopamine at a fixed GNR concentration (0.2 μg/mL). 1:0.01, 1:0.2, 1:0.5, and 1:1 indicate the mass ratio of SGNRs to the dopamine concentrations.

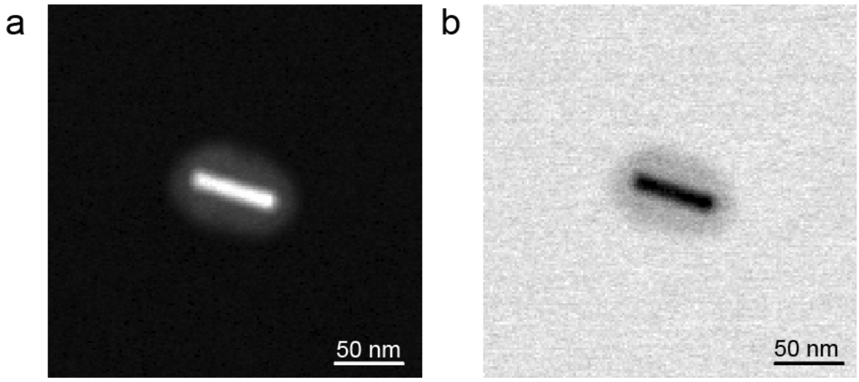

Figure S2.
HAADF and bright field image of SGNR@PDA which had PDA thickness of 25 nm, confirming the core-shell nanostructure of SGNR-polydopamine nanohybrids.

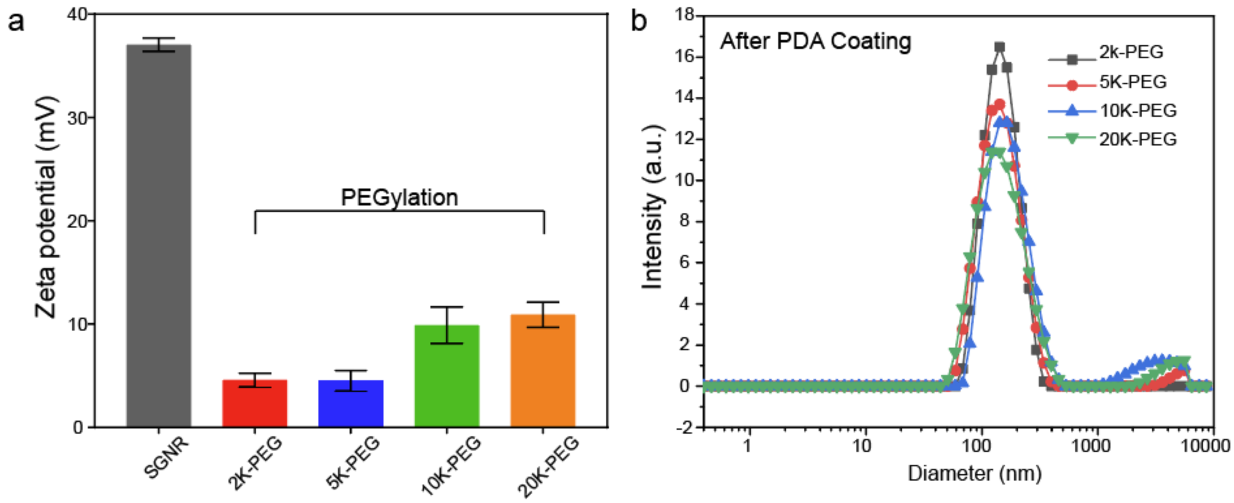

Figure S3.

(a) SGNRs with the size of 51.1 ± 4.2 nm (length) × 7.2 ± 1.2 nm (width) were PEGylated with different molecular weights (e.g., 2k, 5k, 10k and 20k) of methoxy PEG thiol, thus decreasing zeta potential to 4.6 ± 0.6 mV, to 4.5 ± 0.98 mV, to 9.9 ± 1.8 mV, to 10.9 ± 1.2 mV. (b) Interestingly, PEGylated SGNRs with HS-mPEG$_{2k}$ was highly uniform (PDI < 0.1), while slight particle aggregations occurred when SGNRs were PEGylated with 5k, 10k and 20k molecular weight of HS-mPEG. This result suggests that molecular weight of PEG can affect ligand exchange of SGNRs as well as optical PEG configuration for PDA coating.

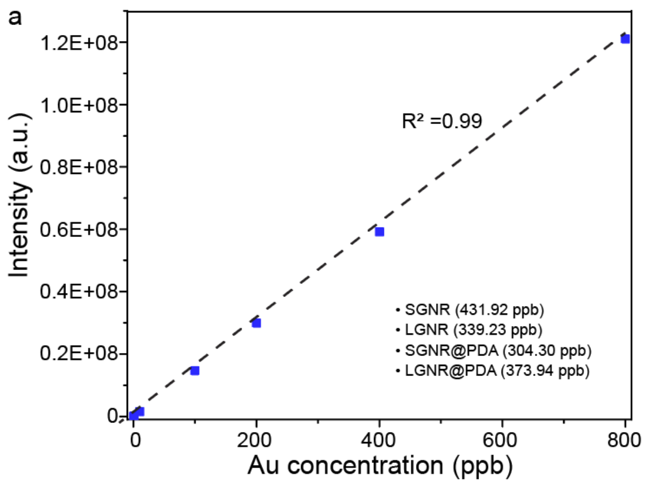

Figure S4.
ICP-MS was used to calculate the Au concentration in each sample. Then, GNR and GNR@PDA concentrations were matched according to the number of Au ions.

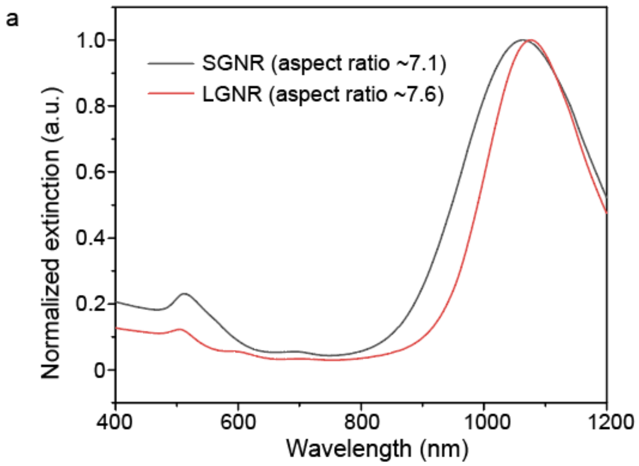

Figure S5.
UV–vis–NIR spectra data of LGNR and SGNR. LGNR and SGNR have longitudinal absorption peaks at 1064 nm and 1076 nm, respectively, due to their high aspect ratio.

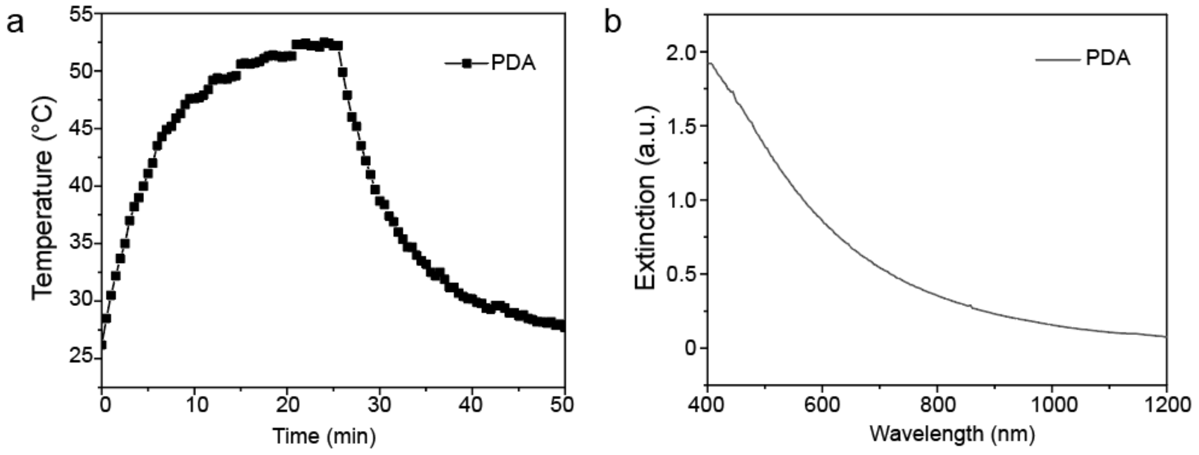

Figure S6.
Temperature curve (a) and UV–vis–NIR spectra (b) of PDA nanoparticles with the size of 100 nm. Photothermal conversion efficiency of PDA nanoparticle was 18% which is significantly lower than GNR-melanin nanohybrids because PDA nanoparticles had low absorption efficiency in NIR-II window as observed in UV–vis–NIR spectra. The NIR-II laser turned off after 25 min of irradiation, and the cooling rate was recorded every 30 s to measure photothermal conversion efficiency.

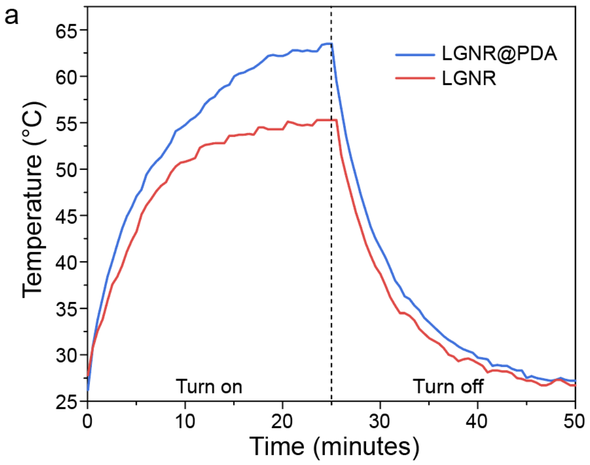

Figure S7.
Temperature curves of LGNR and LGNR@PDA for 50 min under NIR-II (1064 nm) laser irradiation. The temperature of LGNR@PDA at 25 min of irradiation was 7 °C higher than LGNR indicating that PDA coating enhances photothermal performance of LGNR.

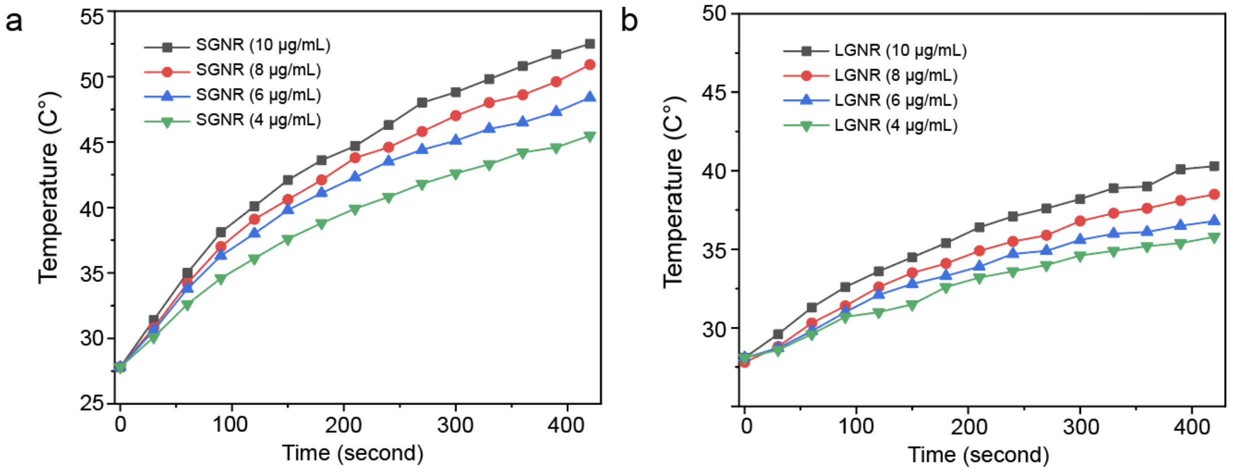

Figure S8.
Temperature curves of SGNR and LGNR with the elevated concentrations (e.g., 4, 6, 8, and 10 μg/mL) for 7 min at 1064 nm laser irradiation. These results indicate that increases in temperature are concentration-dependent due to improved optical absorption.

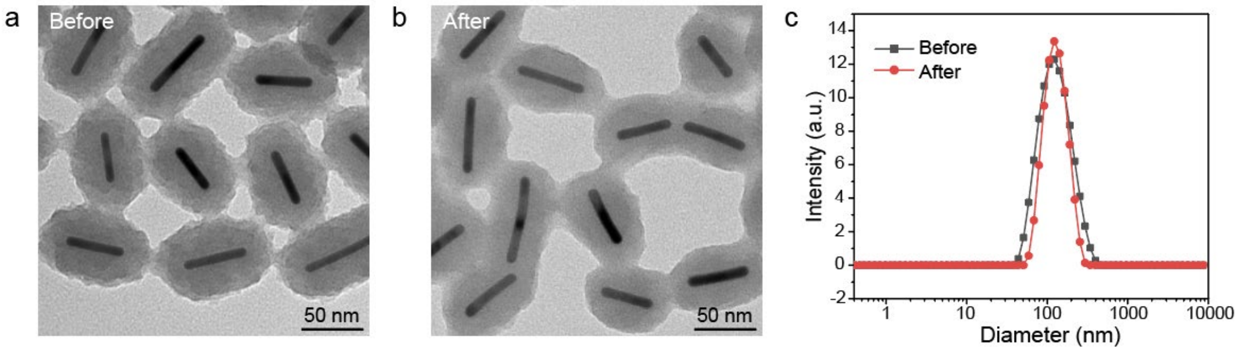

Figure S9.
Robust SGNR@PDA before and after four cycles of 1064 nm laser irradiation. TEM images before (a) and after (b) four cycles of irradiation. (c) Negligible aggregation or disassembly of PDA coating was observed in DLS measurements.

a

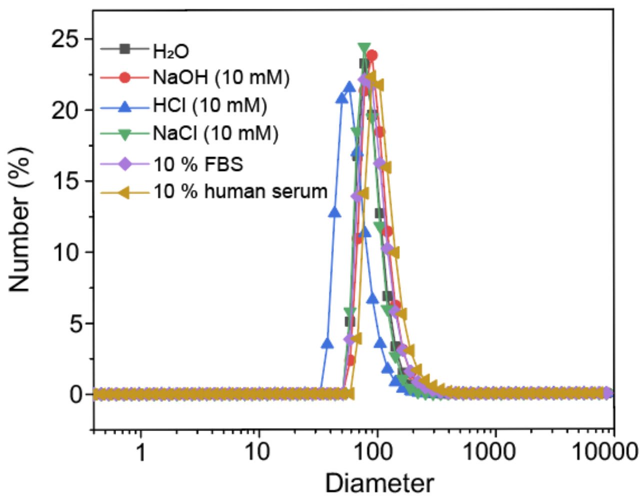

Figure S10.
The colloidal stability of SGNR@PDA in different media (10 mM of HCl (~ pH 2), 10 mM of NaOH (~ pH 12), 10 mM of NaCl, DMEM of 10 % fetal bovine serum (FBS), and the human serum of 10 %). DLS data showed SGNR@PDA had high colloidal stability under different biological and extreme conditions. No disassembly of PDA coating or negligible aggregation was found, indicating that PDA coating is robust and stable.

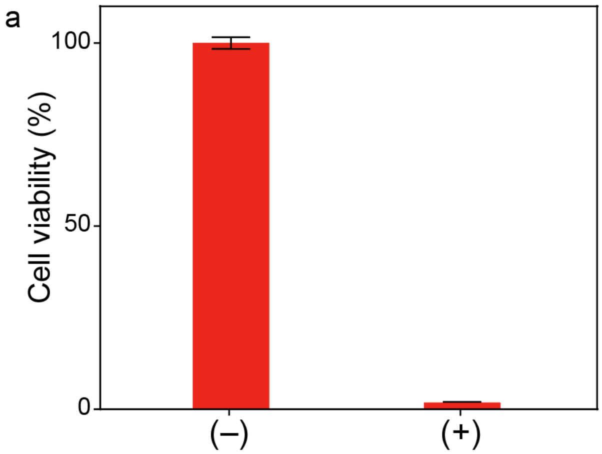

Figure S11.
Photobleaching is known to kill SKOV3 cancer cells. We conducted photobleaching for positive and negative controls, confirming healthy (without photobleaching) and dead SKOV3 (with photobleaching) cancer cells.

Photothermal conversion efficiency

The photothermal conversion efficiency of GNR and GNR@PDA was calculated based on the reported method.[1] The detailed information is described as follows.

The total energy input and heat dissipation from the system is

$$\sum_i m_i C_{p,i} \frac{dT}{dt} = Q_{NP} + Q_{sys} - Q_{diss} \quad \text{---- (1)}$$

where m and C_p is mass, and heat capacity of system components (i) including nanoparticles (NP, e.g., GNR, GNR@PDA), solvent (1.0 mL), and the quartz cuvette (5.66 g). T is temperature of the solution, Q_{NP} is the energy input from the nanoparticles, Q_{sys} is the energy input from the cuvette containing solvent without any nanoparticles, and Q_{diss} is heat dissipation from the system to the surroundings.

The energy input from the nanoparticle (Q_{NP}) can be described as

$$Q_{NP} = I(1 - 10^{-A_\lambda})\eta \quad \text{---- (2)}$$

where I is an incident laser power (W), A_λ is the absorbance of nanoparticles at the wavelength of laser (λ), and η is the photothermal transduction efficiency.

The heat dissipation from the system (Q_{diss}) can be described as

$$Q_{diss} = hS(T - T_{surr}) \quad \text{---- (3)}$$

Where h is heat transfer coefficient, S is the exposed surface area of the quartz cuvette (1 cm^2), and T_{surr} is surrounding temperature.

When temperature rises to an equilibrium (T_{max}), $\frac{dT}{dt} = 0$.

Therefore, the equation (1) becomes

$$Q_{NP} + Q_{sys} = Q_{diss} = hS(T - T_{surr}) \quad \text{---- (4)}$$

When the laser is turned off, the heat input terms ($Q_{NP} + Q_{sys}$) becomes zero. Therefore, the equation (1) becomes

$$\sum_i m_i C_{p,i} \frac{dT}{dt} = -Q_{diss} = -hS(T - T_{surr}) \quad \text{---- (5)}$$

Then, rearranged to equation (6)

$$dt = -\frac{\sum_i m_i C_{p,i}}{hS} \frac{dT}{(T - T_{surr})} \quad \text{---- (6)}$$

Then, the equation (6) integrated to become (7)

$$t = -\frac{\sum_i m_i C_{p,i}}{hS} \ln \frac{(T - T_{surr})}{(T_{max} - T_{surr})} \quad \text{---- (7)}$$

Then equation (7) can be rewritten as

$$t = -\tau_s \ln \theta \qquad \text{---- (8)}$$

$\tau_s = \frac{\sum_i m_i C_{p,i}}{hS}$ (the system time constant for heat transfer) and $\theta = \frac{(T - T_{surr})}{(T_{max} - T_{surr})}$ (dimensionless).

Herein, the system time constant for heat transfer can be calculated by linear regression of the time points (t) versus the negative natural logarithm of θ.

hS can be measured by the cooling rate after the laser turned off. Q_{sys} can be calculated based on the following equation (9):

$$Q_{sys} = hS(T_{max,water} - T_{surr}) \qquad \text{---- (9)}$$

Finally, photothermal efficiency (η) of nanoparticles can be calculated by following equation:

$$\eta = \frac{hS(T_{max} - T_{surr}) - Q_{diss}}{I(1 - 10^{-A_\lambda})}$$

REFERENCES

1. Zhou, J.; Jiang, Y.; Hou, S.; Upputuri, P. K.; Wu, D.; Li, J.; Wang, P.; Zhen, X.; Pramanik, M.; Pu, K., Compact plasmonic blackbody for cancer theranosis in the near-infrared II window. *Acs Nano* **2018,** *12* (3), 2643-2651.
